# Bacteria in Milk

**Published:** 1891-11

**Authors:** 


### BACTERIA IN MILK.

A great deal of the bad milk sent to market is due to natural causes, for which the farmer is not always responsible, but the recent investigations and experiments with bacteria in milk are such as make it plain for most dairymen to avoid a great deal of the unhealthful milk. Every one who handles milk for market should have at least an intelligent understanding of the nature of bacteria, or microbes, as they are called. They exist in air water, soil, and animal and vegetable life.

Cold destroys them; heat develops them, and as a result they are found in milk in a dangerous condition during hot weather. A high temperature of heat will kill them, but it takes a greater amount to kill their spores, which in time become as active as the microbes. Milk contains several species of bacteria, and they have different actions upon the milk and cream. It is due to their development that the cream is "ripened" and that milk sours and curdles. Bacteria require time for their development, and hence cream or milk that has stood for some time abounds the most in these microbes. They collect in the cracks and crevices of cans where the cream or grease has been allowed to stand, and they multiply so rapidly that they soon become dangerous. Milk poured into such cans will become contaminated, and will communicate the poison to other cans into which it may subsequently be poured. In this way poisoned milk spreads rapidly, and causes no end of mischief. Dirt and uncleanliness are especially favorable to the growth of bacteria, and cans that are not properly cleaned, as the milk is poured out of them, will become great breeding places for the germs. Milk cans that are returned from the city on hot days will invariably abound in microbes, for they are seldom cleaned out before they are reshipped. After they are received they must be thoroughly cleansed and purified before other milk is poured into them. The dairyman gets all the blame for impure milk, and hence he should exercise great carefulness. He has everything to lose.—*Ex.*

